# Nerve Sheath Tumors in Neurofibromatosis Type 1: Assessment of Whole-Body Metabolic Tumor Burden Using F-18-FDG PET/CT

**DOI:** 10.1371/journal.pone.0143305

**Published:** 2015-12-01

**Authors:** Johannes Salamon, László Papp, Zoltán Tóth, Azien Laqmani, Ivayla Apostolova, Gerhard Adam, Victor F. Mautner, Thorsten Derlin

**Affiliations:** 1 Department of Diagnostic and Interventional Radiology, University Medical Center Hamburg-Eppendorf, Hamburg, Germany; 2 Mediso Medical Imaging Systems, Budapest, Hungary; 3 Scanomed Ltd., Budapest, Hungary; 4 Department of Radiology and Nuclear Medicine, Otto-von-Guericke University, Magdeburg, Germany; 5 Department of Neurology, University Medical Center Hamburg-Eppendorf, Hamburg, Germany; 6 Department of Nuclear Medicine, Hannover Medical School, Hannover, Germany; National Cancer Center, JAPAN

## Abstract

**Purpose:**

To determine the metabolically active whole-body tumor volume (WB-MTV) on F-18-fluorodeoxyglucose positron emission tomography/computed tomography (F-18-FDG PET/CT) in individuals with neurofibromatosis type 1 (NF1) using a three-dimensional (3D) segmentation and computerized volumetry technique, and to compare PET WB-MTV between patients with benign and malignant peripheral nerve sheath tumors (PNSTs).

**Patients and Methods:**

Thirty-six NF1 patients (18 patients with malignant PNSTs and 18 age- and sex-matched controls with benign PNSTs) were examined by F-18-FDG PET/CT. WB-MTV, whole-body total lesion glycolysis (WB-TLG) and a set of semi-quantitative imaging-based parameters were analyzed both on a per-patient and a per-lesion basis.

**Results:**

On a per-lesion basis, malignant PNSTs demonstrated both a significantly higher MTV and TLG than benign PNSTs (*p* < 0.0001). On a per-patient basis, WB-MTV and WB-TLG were significantly higher in patients with malignant PNSTs compared to patients with benign PNSTs (*p* < 0.001). ROC analysis showed that MTV and TLG could be used to differentiate between benign and malignant tumors.

**Conclusions:**

WB-MTV and WB-TLG may identify malignant change and may have the potential to provide a basis for investigating molecular biomarkers that correlate with metabolically active disease manifestations. Further evaluation will determine the potential clinical impact of these PET-based parameters in NF1.

## Introduction

Neurofibromatosis type 1 (NF1) is a rare hereditary tumor predisposition syndrome caused by a germline mutation in the NF1 tumor suppressor gene [1]. Individuals with NF1 may develop a variety of benign and malignant tumors, the most frequent being peripheral nerve sheath tumors (PNSTs) [2]. PNSTs are neoplasms arising from Schwann cells [3]. So called plexiform neurofibromas may undergo transformation into malignant peripheral nerve sheath tumors (MPNSTs) [4]. Patients with NF1 have a lifetime risk of up to 10% for developing MPNSTs [5,6]. Prognosis is usually poor in these rapidly metastasizing neoplasms, underlining the need for early detection of malignant transformation and identification of risk factors.

F-18-fluorodeoxyglucose (F-18-FDG) positron emission tomography/computed tomography (PET/CT) has become an established and well studied imaging method for the detection of MPNSTs in individuals with NF1 by measuring consumption of radioactively labelled glucose [7–12]. It provides unique insights into the biology of nerve sheath tumors. MPNSTs are highly aggressive neoplasms with marked metabolic activity [9–11]. However, benign plexiform neurofibromas may also exhibit increased metabolic activity in a substantial number of NF1 individuals [7,8]. Not all patients with plexiform neurofibromas will demonstrate metabolically active tumors, and the number (and therefore the volume) of tumors with F-18-FDG uptake differs considerably from case to case [8].

Recently, newer F-18-FDG PET/CT-based volumetric parameters such as metabolic tumor volume (MTV) and total lesion glycolysis (TLG) have gained increasing interest in a variety of cancers [12–18].

The internal tumor burden in individuals with NF1 has been studied using T2-weighted whole-body MRI segmentation techniques [19,20]. High internal tumor load on MRI has been found to be associated with a higher risk of developing MPNSTs in previous studies [21], but not with genetic alterations or serum markers [1,22]. It is an inherent limitation of MRI that it cannot discriminate between tumors which are metabolically active and those who are not [10]. Compared to MRI, PET has the advantage of precise quantitation of tumor metabolic activity in terms of standardized uptake values (SUVs) which can easily be used as cut-off values for differentiating between malignant and benign lesions, e.g. a cut-off > 3.5 usually suggests malignancy [8]. Another advantage of PET/CT over MRI is that an unremarkable investigation excludes malignant change in neurofibromas with a negative predictive value of 100% [8]. There are no parameters on MRI to reliably exclude malignant change [10]. In addition, PET/CT provides a highly sensitive whole-body staging, especially for patients with typical osseous and pulmonary metastases. Differentiation of metastases from benign neurofibromas is challenging if not impossible on MRI. Concerning whole-body imaging, assessment of not just the mere anatomical tumor load but of the metabolically active portion of tumors may provide more useful information concerning NF1 biology, the risk of malignancy or correlation with serum markers.

Therefore, the aim of this study was to determine the metabolically active whole-body tumor burden on F-18-FDG PET/CT in individuals with NF1 using a three-dimensional (3D) segmentation and computerized volumetry technique, and to compare PET WB-MTV, WB-TLG and other imaging-based parameters between patients with benign and malignant PNSTs.

## Patients and Methods

### Patients

The study group consisted of 36 age- and sex-matched patients (20 men; 16 women; age, 36.6 ± 12.3 years; range 16.5 to 68.7 years) with NF1 and benign (*n* = 18) or malignant (*n* = 18) PNSTs who had been referred for an F-18-FDG PET/CT scan for exclusion of MPNSTs between December 2006 and May 2014. Subjects were selected according to the following inclusion and exclusion criteria:

Inclusion:

(I1) Fulfillment of the National Institutes of Health (NIH) diagnostic criteria for NF1 [23].
(I2) Symptomatic lesions or lesions with change in size or texture
(I3) Histopathology and/or clinical/radiological follow-up > 12 months available in patients with focally increased tracer uptake

Exclusion:

(E1) Inability or unwillingness to provide informed consent for the retrospective analysis of the data.
(E2) Patients with atypical NF
(E3) Patients with metastases of MPNST

The study protocol has been approved by the local Clinical Institutional Review Board (Ethic committee of the medical chamber of Hamburg) and complied with the Declaration of Helsinki. All subjects had given written informed consent for the retrospective evaluation of their data. For minors / children we obtained written informed consent from their legal guardians.

### PET/CT Acquisition and Image Reconstruction

F-18-FDG PET/CT images were obtained using a Gemini GXL10 scanner (Philips Medical Systems, Best, The Netherlands). Patients fasted for at least 6 h before injection of 350 MBq of F-18-FDG. During the uptake period of about 60 min, patients received oral contrast (20 ml Gastrolux CT^®^ in 1 l water; Sanochemia Diagnostics, Neuss, Germany). Imaging started with a low-dose CT of the whole body (120 kV, 80 mA, transaxial FOV 600 mm, no gap, collimation 10 x 1.5 mm, pitch 1.1, rotation time 0.5 s, slice thickness 5 mm, matrix 512 x 512). Then, a total-body emission scan was performed in the caudocranial direction with 90 s per bed position at head and thorax, and 60 s at the legs. Transversal PET slices were reconstructed into a 144 x 144 matrix using the iterative 3DLOR reconstruction algorithm of the system software with standard parameter settings. Spatial resolution in the reconstructed PET images was about 8 mm full-width-at-half-maximum. Transaxial, sagittal, and coronal images and fused images were displayed for review.

### Image Analysis

Image analyses were undertaken on a workstation with a research software package (InterView^™^ FUSION; Mediso Medical Imaging Systems Ltd., Budapest, Hungary).

#### PET image analysis

PET images were visually evaluated for the presence of focal radiotracer uptake above background corresponding to space-occupying lesions on CT images. We herein define all lesions with increased glucose metabolism and therefore significant F-18-FDG uptake (SUV_max_ ≥ 2.0) as metabolically active, i.e. PET positive (PET+). The number of PET+ PNSTs was recorded. Then, automated segmentation of metabolically active tumors in PET+ patients was performed using a stopping rule of 2.0, 2.5 and 3.5 [24]. These established cut-off values were chosen to assess likely malignant lesions (SUV_max_ ≥ 3.5), additional equivocal lesions requiring follow-up (SUV_max_ ≥ 2.5) [7,8,10], and finally all metabolically active lesions (SUV_max_ ≥ 2.0). The voxels of normal organs such as heart, liver, kidneys, ureters, and bladder, as well as false-positive lesions such as inflammatory lesions were manually excluded. Semiquantitative analysis was performed by obtaining the mean and maximum standardized uptake values (SUV_mean_, SUV_max_) of each lesion. The standardized uptake value (SUV) was calculated using the following formula: tissue concentration (MBq/g)/injected dose (MBq)/body weight (g). The metabolic tumor volume (MTV) of each lesion was measured and added to the whole-body MTV (WB-MTV). The total lesion glycolysis (TLG) was calculated as (MTV) × (mean SUV) [14]. Whole-body total lesion glycolysis (WB-TLG) was calculated by summing up the individual TLG of each lesion.

#### CT image analysis

The largest diameter of each lesion was determined on the corresponding coregistered PET/CT images.

### Reference standard and histopathological evaluation

All patients with PNSTs with increased tracer uptake underwent histopathological evaluation [25] and/or clinical and radiological follow-up > 12 months.

### Statistical analysis

Continuous variables are expressed as mean ± SD. Categoric variables are presented with absolute and relative frequencies. Analyses were performed both on a per-lesion and a per-patient basis. MTV, TLG and a set of other imaging-derived parameters (maximum SUV_mean_, maximum SUV_max_, maximum diameter) were recorded for all benign and malignant lesions, and compared. WB-MTV, WB-TLG and other imaging-derived parameters were also compared between patient groups with benign and malignant PNSTs. For between-group comparisons of continuous data, *P* values were calculated from Mann-Whitney U rank sum tests. Receiver operating characteristic (ROC) analysis was performed to define the optimum cut-off value for SUV_max_, MTV and TLG. Statistical significance was established for *P* values of less than 0.05. Statistical analysis was performed using GraphPad Prism 5.0^®^ for Windows (GraphPad Software In., La Jolla, Calif, USA).

## Results

Evaluation of F-18-FDG PET/CT scans was feasible in all patients. The MPNST group consisted of 18 patients (10 men, 8 women; mean age, 35.8 ± 12.9 years; range, 17.1–68.7 years). The control group matched for gender and age consisted of 18 patients (10 men, 8 women; mean age, 37.4 ± 12.1 years; range, 16.5–63.2 years).

19 MPNSTs were confirmed by histopathological evaluation in 18 patients. 14 other tumors were found to be benign by histology. All other PET+ lesions were classified as benign after a mean follow-up of 54 ± 23 months (range, 13–98).

### Lesion-based analysis

**Fig 1:**
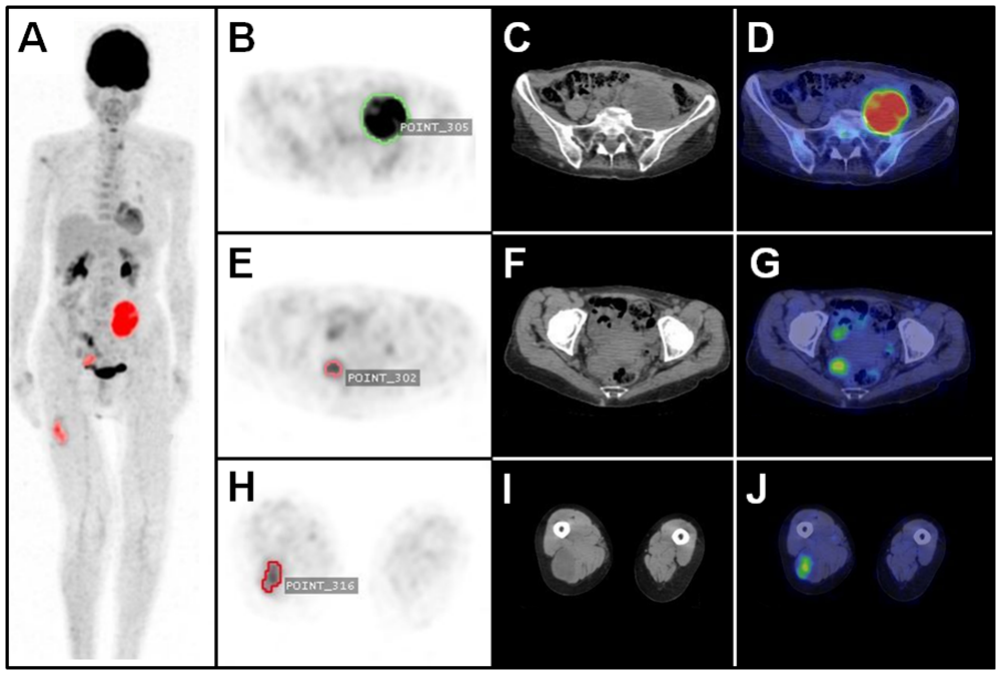
Automated 3D-segmentation and volumetric assessment of nerve sheath tumors. Maximum intensity projection PET image (**A**) with automatically delineated metabolically active tumors (*red*). Axial PET image (**B**) with volumetric ROI (*green*) of a MPNST (SUV_max_ / MTV / TLG: 11.2 / 182592 / 894700) in the left pelvis; corresponding CT (**C**) and fused PET/CT image (D). Axial PET image (**E**) with volumetric ROI (*light red*) of a plexiform neurofibroma (PNF) (SUV_max_ / MTV / TLG: 4.7 / 14848 / 41574) close to the rectum; corresponding CT (**F**) and PET/CT image (**G**). Axial PET image (H) with volumetric ROI (*dark red*) of a BPNST (SUV_max_ / MTV / TLG: 3.7 / 15872 / 41267) in the right thigh; corresponding CT (**I**) and PET/CT image (**J**).

An example of a volumetric analysis in a NF 1 patient with PNSTs is shown in Fig 1. 74 metabolically active lesions were found in 34 (94.4%) patients at a SUV_max_ threshold of 2.0, and therefore considered as PET+. The mean SUV_max_ of PET+ lesions was 5.8 ± 3.5 (range, 2.2–16). The average SUV_mean_ of these lesions was 3.1 ± 1.0 (range, 1.7–6.7). The mean size of PET+ tumors was 62.1 ± 49.2 mm (range, 11–292 mm).

**Fig 2:**
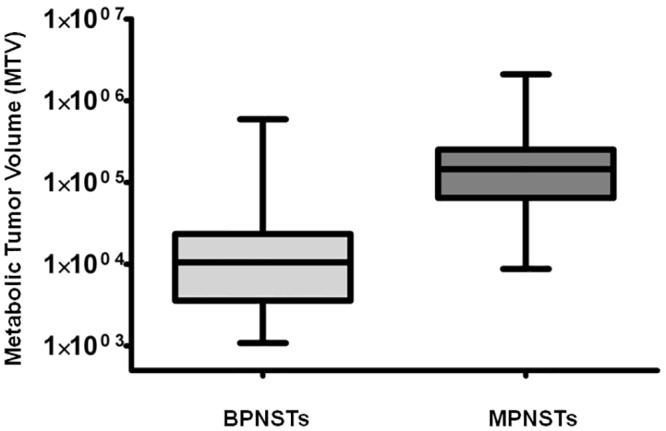
Comparison between MTV of BPNSTs versus MPNSTs on a per-lesion basis (SUV_max_ threshold 2.0). MTV is significantly higher in MPNSTs (*p* < 0.0001).

**Fig 3:**
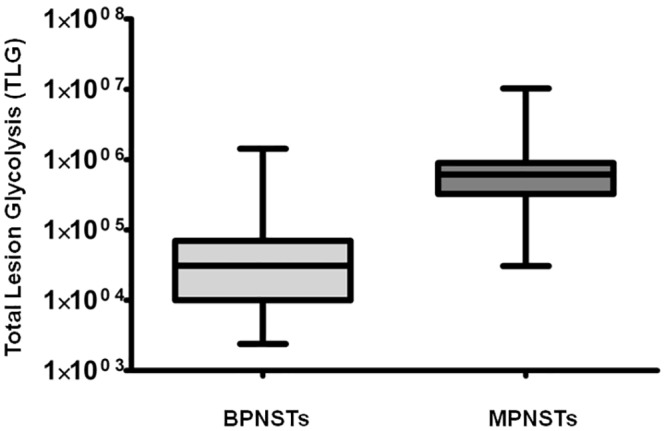
Comparison between TLG of BPNSTs and MPNSTs on a per-lesion basis (SUV_max_ threshold 2.0). TLG is significantly higher in MPNSTs (*p* < 0.0001).

Detailed data on metabolic tumor volume and total lesion glycolysis at different SUV_max_ thresholds are shown in Table 1. Box plots demonstrating the significant difference between MTV and TLG of MPNSTs versus BPNSTs are shown in Figs 2 and 3.

**Table 1:** Per-lesion analysis of metabolic tumor volume and total lesion glycolysis of benign vs. malignant PNSTs at different SUV_max_ thresholds.

	SUVmax > 2.0		*p*	SUVmax > 2.5		*p*	SUVmax > 3.5		*p*
	BPNSTs	MPNSTs		BPNSTs	MPNSTs		BPNSTs	MPNSTs	
MTV (mm³)									
Mean ± SD	29547 ± 81432	262383 ± 460991	< 0.0001*	12957 ± 14562	207581 ± 484072	< 0.0001*	7626 ± 8174	144368 ± 287296	< 0.0001*
Range	1088–594432	8768–2101568		1024–56576	8768–1758500		1024–34560	2304–1304704	
TLG									
Mean ± SD	81988 ± 201163	1135248 ± 2258235	< 0.0001*	44679 ± 54981	954137 ± 2105349	< 0.0001*	33839 ± 41122	873232 ± 1788102	< 0.0001*
Range	2393–1426636	30688–10297683		2764–243267	31564–9495900		3891–179712	8985–8089164	

MTV- metabolic tumor volume; TLG—total lesion glycolysis; BPNST—benign peripheral nerve sheath tumors; MPNST—malignant peripheral nerve sheath tumors; SD—standard deviation;

*—statistically significant

**Fig 4:**
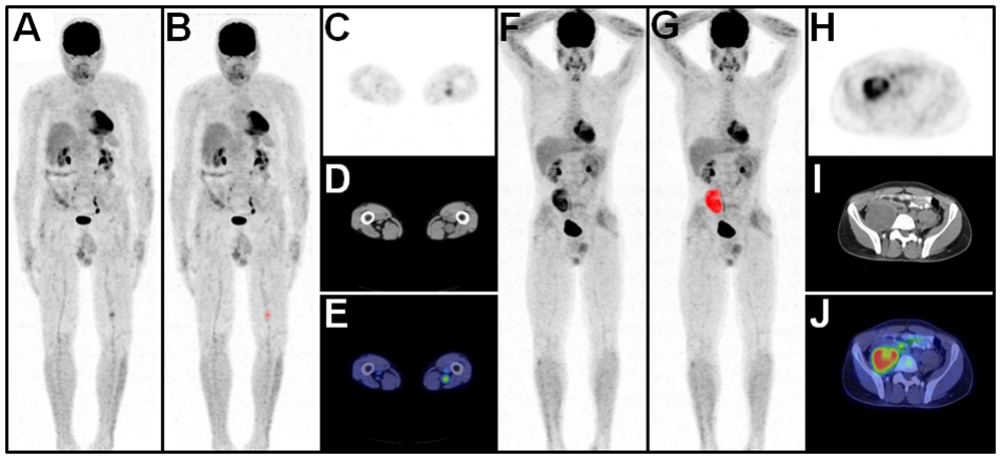
Solitary BPNST versus solitary MPNST. Maximum intensity projection PET image (**A**) of a patient with a solitary BPNST (SUV_max_ / MTV / TLG: 2.6 / 2885 / 6488) in the left thigh with automatically delineated metabolically active tumors (*red*) (**B**), corresponding axial PET (**C**), CT (**D**) and fused PET/CT image (**E**). Maximum intensity projection PET image of a patient with a solitary MPNST (SUV_max_ / MTV / TLG: 7.1 / 171328 / 633913) in the right pelvis (**F**) with automatically delineated metabolically active tumors (*red*) (**G**) with corresponding PET (**H**), CT (**I**) and PET/CT image (**J**).

Compared to BPNSTs, MTV and TLG of MPNSTs were significantly higher at SUV_max_ thresholds of 2.0, 2.5 and 3.5, (*p* < 0.0001). Compared to BPNSTs, MPNSTs also demonstrated a significantly higher SUV_max_, SUV_mean_, and tumor diameter (*p* < 0.0001 in all cases). Examples of a solitary MPNST and a solitary BPNST are shown in Fig 4.

Detailed data (per-lesion analysis) on imaging-derived parameters of malignant and benign PNSTs are shown in Table 2.

**Table 2:** Data on imaging-derived parameters of benign and malignant PNSTs at a SUV_max_ threshold of 2.0.

Parameter	NF1		*p*
	BPNSTs	MPNSTs	
Number of (PET+) lesions (n)			
	55	19	
Tumor size (mm)			
Mean ± SD	46 ± 30	114 ± 61	< 0.0001*
Range	11–172	52–292	
SUV_max_			
Mean ± SD	4.2 ± 1.6	10.3 ± 4.2	< 0.0001*
Range	2.2–9.1	4.6–16	
SUV_mean_			
Mean ± SD	2.7 ± 0.5	4.2 ± 1.2	< 0.0001*
Range	1.7–3.9	2–6.7	
MTV (mm³)			
Mean ± SD	29547 ± 81432	262383 ± 460991	< 0.0001*
Range	1088–594432	8768–2101568	
TLG			
Mean ± SD	81988 ± 201163	1135248 ± 2258235	< 0.0001*
Range	2393–1426636	30688–10297683	

MTV- metabolic tumor volume; TLG—total lesion glycolysis; BPNST—benign peripheral nerve sheath tumors; MPNST—malignant peripheral nerve sheath tumors; SD—standard deviation;

*—statistically significant.

Neither MTV nor TLG were significantly correlated with gender or age at all investigated SUV_max_ thresholds on a per-lesion basis (*p* > 0.05 in all cases).

### Patient-based analysis

34 (94.4%) of 36 NF1 patients demonstrated metabolically active PNSTs. The mean WB-MTV per patient was 212040 ± 282687 mm^3^ (range, 2880–2107008 mm^3^). Compared to patients with only BPNSTs, the WB-MTV was significantly higher in patients with MPNSTs at all SUV_max_ thresholds (*p* < 0.001 in all cases). The mean WB-TLG per patient was 814423 ± 1801860 mm^3^ (range, 7488–10315091 mm^3^). Patients with MPNSTs had a significantly higher WB-TLG at all SUV_max_ thresholds (*p* < 0.001). Neither WB-MTV nor WB-TLG were significantly correlated with gender or age at all investigated SUV_max_ thresholds on a per-patient basis (*p* > 0.05 in all cases).

**Fig 5:**
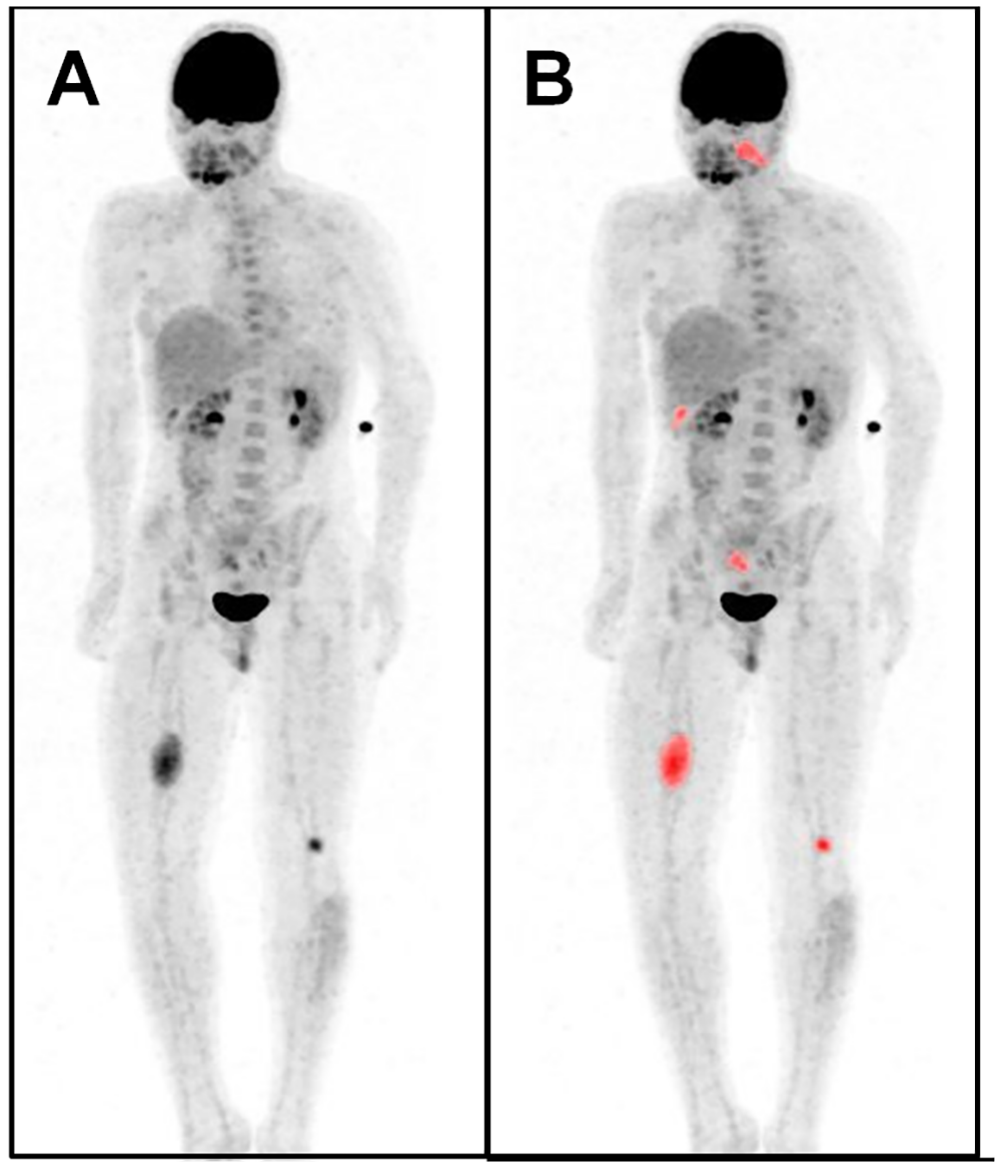
Multiple metabolically active tumors. Maximum intensity projection PET image (**A**) of a patient with a MPNST in the right upper leg (SUV_max_ / MTV / TLG: 5.3 / 63488 / 190464), plexiform neurofibromas in the left head and neck area (SUV_max_ / MTV / TLG: 3.6 / 17088 / 42720), below the right liver lobe (SUV_max_ / MTV / TLG: 3.7 / 3584 / 8960), in the right pelvis (SUV_max_ / MTV / TLG: 2,5 / 6435 / 14157) and in the left upper leg (SUV_max_ / MTV / TLG: 5.3 / 4096 / 13107). Automatically delineated metabolically active tumor volumes (*red*) (**B**).

Detailed data (per-patient analysis) on WB-MTV and WB-TLG at different SUV_max_ thresholds are shown in Table 3. Data (per-patient analysis) on other imaging-derived parameters comparing the patient group with only BPNSTs and the patient group diagnosed with malignant and benign PNSTs are shown in Table 4. Fig 5 shows a MPNST patient with multiple metabolically active tumors.

**Table 3:** Metabolic tumor volume and total lesion glycolysis of benign and malignant PNSTs at different SUV_max_ thresholds (per-patient analysis).

	SUVmax > 2.0		*p*	SUVmax > 2.5		*p*	SUVmax > 3.5		*p*
	BPNST Group	MPNST Group		BPNST Group	MPNST Group		BPNST Group	MPNST Group	
MTV (mm³)									
Mean ± SD	933348 ± 182184	309786 ± 476008	< 0.0001*	26710 ± 30288	229857 ± 401815	< 0.0001*	14896 ± 12901	165380 ± 299704	0.0003*
Range	2880–717056	83456–2107008		1024–104256	21696–1761444		2432–34624	11712–1306560	
TLG									
Mean ± SD	241878 ± 446054	1259842 ± 2299646	0.0002*	91295 ± 106409	1100238 ± 2205196	< 0.0001*	40251 ± 48086	924919 ± 1883390	0.0008*
Range	7488–1760217	30668–10315091		2764–350348	31564–9511797		3891–179712	15859–8097516	

MTV- metabolic tumor volume; TLG—total lesion glycolysis; BPNST—benign peripheral nerve sheath tumors; MPNST—malignant peripheral nerve sheath tumors; SD—standard deviation;

*—statistically significant

**Table 4:** Data on imaging-derived parameters comparing the patient group with benign PNST only and the group with benign and malignant PNSTs at a threshold of 2.0.

Parameter	NF1		*p*
	BPNST Group	MPNST Group	
Number of lesions (n)			
Mean ± SD	2.1 ± 2.2	2.3 ± 1.6	0.29
Range	1–9	1–7	
Tumor size (mm)			
Mean ± SD	52.6 ± 35.4	86.6 ± 56.2	0.002*
Range	11–172	12–292	
SUV_max_			
Mean ± SD	3.9 ± 1.4	7.2 ± 4	0.002*
Range	2.2–9.1	2.3–16	
SUV_mean_			
Mean ± SD	2.6 ± 0.5	3.5 ± 1.1	0.003*
Range	1.7–3–8	2–6.7	

BPNST—benign peripheral nerve sheath tumors; MPNST—malignant peripheral nerve sheath tumors; SD—standard deviation;

*—statistically significant.

### ROC analysis of MTV and TLG for identification of malignancy

#### Per-patient basis

Both a WB-MTV cut-off value and a WB-TLG cut-off value could differentiate between patients with MPNSTs and those with only BNSTs at all SUV_max_ thresholds (*p* < 0.001 in all cases).

#### Per-lesion basis

Both a MTV cut-off value and a TLG cut-off value could differentiate between patients with MPNSTs and those with only BNSTs at all SUV_max_ thresholds (*p* < 0.0001 in all cases). The optimum cut-off-value for SUV_max_ was > 5.5 (AUC 0.96, *p* < 0.0001), yielding a sensitivity of 95% and a specificity of 85% for differentiation of malignant and benign lesions. The optimum cut-off-value for the TTL ratio was > 2.6 (AUC 0.98, *p* < 0.0001), resulting in a sensitivity of 100% and a specificity of 87%.

Results of the ROC analysis are summarized in Table 5.

**Table 5:** ROC analysis for MTV and TLG on both a per-lesion and a per-patient basis.

	MTV					
	SUV_max_ threshold	Sensitivity (%)	Specificity (%)	MTV (mm^3^)	AUC	p
Per -lesion	2.0	95	74.1	> 22336	0.92	<0.0001
	2.5	95	80.5	> 19680	0.94	<0.0001
	3.5	84.2	72	> 10848	0.90	<0.0001
Per- patient	2.0	100	86.7	> 82400	0.92	<0.0001
	2.5	93.7	78.6	> 42528	0.93	<0.0001
	3.5	94.1	75	> 15648	0.95	<0.0003
	TLG					
	SUV_max_ threshold	Sensitivity (%)	Specificity (%)	TLG	AUC	p
Per -lesion	2.0	94.7	78	> 99213	0.94	<0.0001
	2.5	94.1	83	> 82627	0.96	<0.0001
	3.5	89	71	> 43392	0.90	<0.0001
Per- patient	2.0	94.4	80	> 204614	0.88	<0.0001
	2.5	94	79	> 133235	0.93	<0.0001
	3.5	88.2	75	> 102006	0.91	<0.001

## Discussion

In this study, we assessed the metabolically active whole-body tumor burden in NF1 patients using F-18-FDG PET/CT. In a cohort matched for sex and age, we could introduce WB-MTV and WB-TLG as novel parameters for biological characterization of NF1 patients. Most importantly, we could demonstrate that these parameters are significantly different between malignant and benign lesions (*p* < 0.0001), and that MPNST patients have significantly different whole-body MTV and TLG compared to patients with only benign tumors (*p* < 0.001).

F-18-FDG PET/CT has become an established and powerful tool for detection of malignant peripheral nerve sheath tumors in patients with NF1 [7,8,11]. In the past, various F-18-FDG PET/CT imaging parameters have been explored in order to further characterize PNSTs and to facilitate sensitive detection of MPNSTs. Among all PET derived parameters, SUV_max_ represents the most commonly used parameter for detecting MPNSTs [26]. Recommended cut-off values range from 3.5 to 6.1 [26,27], but a SUV_max_ of 3.5 is the most widely used cut-off value [7,8,26]. In this study, the optimum SUV_max_ cut-off value would have been ≥ 4.5, yielding maximum sensitivity and a specificity of 71%. However, SUV_max_ only represents the highest metabolic activity of a pixel within the tumor, not taking into account other biological parameters such as the tumor volume. MPNSTs often arise from so called plexiform neurofibromas which are often very large and -due to necrosis and fibrotic scar tissue- markedly heterogeneous tumor manifestations with highly variable shapes [4,26,28]. Therefore, one-dimensional assessments such as SUV_max_ do not necessarily represent the real tumor burden or tumor biology. To identify malignant change, active tumor volume is more relevant to increase specificity and reduce false positive diagnoses than a single pixel with high metabolic activity. Indeed, MTV and TLG incorporate both metabolic activity and three-dimensional tumor volume. In the present study, both MTV and TLG were significantly different between benign and malignant PNSTs at all SUV_max_ thresholds (*p* < 0.001). ROC analyses revealed that both parameters could be used to identify malignant change with similar sensitivity and specificity like the established SUV_max_ cut-off. However, specificity was not fully satisfying for all assessed parameters (MTV, TLG, SUV_max_), which is a known challenge in MPNST imaging.

With improving image analysis tools and three-dimensional display techniques, volume based parameters of F-18-FDG PET may be evaluated rapidly and consistently [29–33]. However, there is currently no standard methodology for assessment of volumetric PET parameters. Previous studies assessing MTV and TLG in a variety of tumors used different methods to define a threshold for SUVs to delineate the metabolic tumor volume. Some studies also used an isocontour threshold method based on F-18-FDG uptake segmented above a threshold of 2.5 or 3.0 [30,31]. Others assessed MTV using different methods like setting a margin threshold of 50% of SUV_max_ of that lesion [32]. In the present study we used three different fixed SUV_max_ isocontour thresholds of 2.0, 2.5 and 3.5. The rationale for that approach was that a threshold of ≥ 3.5 represents the most commonly used cut-off value for differentiation between MPNSTs and PNSTs [26]. Indeed, all MPNSTs in this study demonstrated a SUV_max_ of at least 4.6. A SUV_max_ threshold of ≥ 2.5 is usually used to define the need for follow-up of PNSTs to rule out malignant transformation in the future [10,26]. The lowest threshold of ≥ 2.0 was chosen to evaluate the volume of all PNSTs with relevant metabolic activity, and was therefore used to define PET+ tumors in this study. A threshold of ≥ 3.5 should be regarded as most important and should remain the basis for future studies, for it can identify malignant tumor tissue with high reliability.

Whole-body MRI is widely used in NF1 to define a subpopulation with internal tumor burden, to further characterize PNSTs and to provide a means for follow-up examinations to identify growing lesions [19–22]. Assessment of whole-body internal tumor burden using segmentation techniques for T2-weighted whole-body MRI sequences has been investigated in several studies [20,21]. Higher internal tumor burden on T2-weighted imaging has been associated with an increased risk for developing MPNSTs. In a study by Nguyen et al., patients with MPNSTs demonstrated a significantly higher whole-body tumor volume compared to NF1 patients without MPNSTs. However, the non-MPNST group included a significant number of patients without internal tumors which have a substantially lower risk for developing malignancy [21]. In the present study, both WB-MTV and WB-TLG were significantly higher in patients with MPNSTs (*p* < 0.001). Given the fact that only a small proportion of internal tumors in NF1 are metabolically active and that MPNSTs uniformly demonstrate high F-18-FDG uptake, assessment of WB-MTV may help to more precisely define populations at risk than mere assessment of morphologic tumor burden on T2w. This is supported by the fact that so far, MRI-derived volumetric assessment showed no correlation with genetic analyses, e.g. large NF1-deletions, even though constitutional large NF1-deletions are generally associated with more severe clinical manifestations [1]. In addition, known serum markers of Schwann cells like S100β have shown no correlation with MRI tumor burden [22]. Assessment of biologically active tumor burden on PET might provide a more promising parameter for correlation analysis, hypothesizing that serum markers of tumor burden may be mainly originating from active tumors. Further studies comparing T2w-derived whole-body tumor burden, WB-MTV and WB-TLG will shed light on the usefulness of these techniques for providing an imaging biomarker correlating with known circulating biomarkers such as adrenomedullin, MIA or SOX9 [34–36].

Some limitations of the present study should be mentioned. First, given the retrospective nature of this study, a selection bias cannot be ruled out. Second, the number of included patients is relatively small. However, the number of included MPNSTs (*n* = 19) is comparable to or higher than in other studies [7,26,27]. A particular strength of this study is that the study population is matched for both age and gender which has not been done in most other studies comparing malignant and benign PNSTs. Third, histopathologic evaluation could not be performed in all PNSTs for both practical and ethical reasons. However, regarding the follow-up of 54 ± 23 months, it is highly unlikely that tumors might have been misclassified. Finally, we did not assess the prognostic significance of MTV and TLG. Although it is tempting to assume that large tumors may carry a worse prognosis, we consider the most important value of PET MTV and TLG in NF1 patients to comprehensively characterize the clinical phenotype and to provide a basis for evaluation of genotype-phenotype relations and biochemical markers.

## Conclusion

WB-MTV and WB-TLG identify a distinct phenotype of neurofibromatosis patients, characterize biologic subpopulations, and may discriminate between malignant and benign PNSTs. Therefore, one might assume that MTV and TLG provide the basis for investigating molecular biomarkers that correlate with metabolically active disease manifestations. Further evaluation will determine the potential clinical impact and prognostic significance of these PET-based parameters in NF1.
